# Association of H-Type Hypertension with miR-21, miR-29, and miR-199 in Kazahks of Xinjiang, China

**DOI:** 10.1155/2022/4632087

**Published:** 2022-09-26

**Authors:** Xin He, RuLin Ma, Yu Li, Haixia Wang, YiZhong Yan, YiDan Mao, ShengYu Liao, XueYing Sun, ShuXia Guo, Heng Guo

**Affiliations:** ^1^Department of Public Health, Shihezi University School of Medicine, North 2th Road, Shihezi 832000, Xinjiang, China; ^2^NHC Key Laboratory of Prevention and Treatment of Central Asia High Incidence Diseases First Affiliated Hospital, School of Medicine, Shihezi University, Shihezi, China

## Abstract

**Objective:**

This study aims to analyze the expressions of miR-21, miR-29, and miR-199 in the serum of the patients with H-type hypertension among Kazakhs. Then, we analyzed the effect of MTHFR 677C > T polymorphism on the association between the above miRNA and H-type hypertension.

**Method:**

In this study, the expression of miR-21, miR-29, and miR-199 was quantitatively measured in 120 serum samples and then stratified according to the C677T polymorphism to analyze the relationship between target miRNAs and HHcy.

**Results:**

The expression of miR-21/-29 in the hypertension group was higher than the normal group (*P* < 0.001). And the expression of miR-199 was higher in the hcy group than in the normal group (*P* < 0.001). In the CC and CT genotypes of MTHFR 677C > T, the expression of miR-21 was lower in the HHcy patients than in the normal individuals (*P* = 0.005 and *P* = 0.001) and miR-199 was significantly higher in the HHcy patients than in the normal ones (*P* = 0.002 and *P* = 0.048). No such difference was found in the TT genotype. Logistic regression analysis showed that after adjusting for sex, age, BMI, systolic blood pressure, diastolic blood pressure, and MTHFRC677 T gene polymorphism, miR-21 was negatively correlated with hcy (OR = 0.222, 95% CI (0.101–0.485), *P* < 0.001) and miR-199 was positively correlated with hcy (OR = 1.823,95%CI (1.272∼2.614), *P* = 0.001).

**Conclusion:**

miR-21, miR-29, and miR-199 are associated with H-type hypertension in the Kazakhs, especially hyperhomocysteinemia. And these three miRNAs may serve as biomarkers to provide clues to the potential pathogenesis of H-type hypertension.

## 1. Introduction

Homocysteine (hcy) is an intermediate product of methionine metabolism, and pathological accumulation causes homocysteinemia (HHcy, hcy ≥15 umol/l) [1]. Studies have shown that HHcy and hypertension are synergistic factors that increase the risk of cardiovascular disease [2]. In China, 75% of people with hypertension have hyperhomocysteinemia (defined as H-type hypertension) [3], but the pathogenesis is unclear. There is growing evidence that genetic factors are involved in the biology of H-type hypertension.

MicroRNAs (miRNAs) are a class of small noncoding RNAs about 22 nucleotides long. By binding to the 3′-untranslated region (3′-UTR) of mRNAs, miRNAs degrade mRNAs and inhibit the translation of target genes and subsequent silencing gene expression, which is the main mode of action of miRNAs. It has received much attention as a stable and specifically expressed marker in cardiovascular diseases, including hypertension [4]. Indeed, there is growing evidence that miR-21, miR-29b, and miR-199a are involved in biological processes associated with H-type hypertension. Located on chromosome 17q23.2 [5], miR-21 has long been valued for its role in tumor, cardiovascular, and lung diseases. MiR-21 acts like a proto-oncogene and promotes malignancy progression by participating in processes such as regulation of the cell cycle and gene expression and has a key role in angiogenesis, apoptosis, and cardiac and renal fibrosis [6]. MiR-21 has been shown to be a key molecule in the regulation of vascular remodeling during essential hypertension [7]. Clinical and experimental studies have demonstrated that miR-21 expression levels are positively correlated with hypertension [8]. The miR-29 family has three members (miR-29a, miR-29b, and miR-29c), and studies have shown that miR-29b plays an important role in cardiac fibrosis (by inhibiting extracellular matrix production), lipid homeostasis, endothelial function (by increasing NO production), left ventricular remodeling, T2DM management, and a variety of cancers [6]. And miR-29b expression levels are positively correlated with 24 h mean blood pressure, daytime mean blood pressure, office blood pressure, and LVMI (left ventricular mass index) [9]. MiR-199a is a new miRNA molecule with multiple biological functions discovered in serum and other tissues in animals in recent years, which plays a key role in the growth and development of many organisms, as well as in the pathophysiology of some diseases, and is particularly closely associated with various tumors, vascular injury, cell proliferation, and regulation. Basic and clinical studies at home and abroad have confirmed that miR-199a is mainly expressed in cardiomyocytes, its expression level is abnormal in a variety of cardiovascular diseases, and its overexpression induces cardiomyocyte-specific miR-199a transgenic mice with cardiac hypertrophy and heart failure [10]. Lynch et al. [11] found a significant negative correlation between miR-199-5p and SMAD4 (recombinant mothers against decapentaplegic homolog 4, which has been previously implicated in hypertension) expression, with miR-199a-5p affecting blood pressure by targeting SMAD4. This study also confirmed that serum miR-199a-5p expression in the patients with methylenetetrahydrofolate reductase (MTHFR) 677TT genotype was somehow associated with hypertension in individuals in the TT group. It is becoming increasingly apparent that these findings highlight the importance of miR-21/-29b/-199a as a potential biomarker for H-type hypertension.

Polymorphisms in genes that code for hcy metabolism-related enzymes can change the result of this metabolic locus, as well as gene and miRNA expression levels [12]. The methylenetetrahydrofolate reductase (MTHFR) C677T polymorphism is thought to be a contributor to hcy and is associated with an increased risk of hypertension [13], with meta-analysis suggesting an increased risk of 24–87% [14]. Therefore, it seems necessary to investigate the relationship between MTHFR gene polymorphisms and miR-21, miR-29b, and miR-199a levels.

No study has considered the effect of the MTHFR genotype on the expression of the above miRNAs. Therefore, the aim of this study was to investigate the association of serum miR-199, miR-29, and miR-21 expression levels with H-type hypertension and to further investigate whether MTHFR genotype affects the association of miRNAs with H-type hypertension, which would provide insights to the underlying pathogenesis of H-type hypertension.

## 2. Methods

### 2.1. Subjects and Sample Collection

Subjects came from the Kazakh cohort of Xinyuan County from 2009 to 2017. The subjects were divided into 4 groups based on serum hcy levels and blood pressure levels: H-type hypertension group (HT+HCY, hypertension accompanied by hyperhomocysteinemia), simple hypertension group (HT, hypertension and no hyperhomocysteinemia), simple hyperhomocysteinemia group (HCY, hyperhomocysteinemia and no hypertension), and normal control group (CONTROL, no hypertension and no hyperhomocysteinemia), with a sample size of 100 subjects in each group. Subsequently, 30 subjects were randomly selected from each group for microRNA measurement. Hyperhomocysteinemia was defined as plasma hcy ≥15 *μ*mol/L; according to the Chinese Guidelines of hypertension management in 2018 [3], those with systolic blood pressure≥140 mm·Hg and/or diastolic blood pressure≥90 mm·Hg; those with a clear previous history of hypertension and diagnosed at a hospital at the county level or above; or those who have taken hypertensive medication in recent 2 weeks were judged to be hypertension.

### 2.2. Blood Biochemistry

5 ml of whole blood was collected from the subject's fasting morning blood with sodium heparin anticoagulation, centrifuged to separate the plasma, numbered, and stored in the refrigerator at −80°C for testing the biochemical indexes.

### 2.3. Genotyping

DNA was extracted in the subject's serum samples using the kit (TIANamp Genomic DNA Kit) and typing of the MTHFR rs1801133 single nucleotide polymorphism was completed using an ABI13730XL sequencer.

### 2.4. Selection of miRNA

The miRNAs were selected by the following inclusion criteria in our study.

All miRNAs are expressed in plasma although they are not unique; they all have a wide distribution on chromosomes.

They have not been studied in H hypertension: miR-21 and miR-29 have been studied in patients with hypertension but not yet in patients with H hypertension; miR-199a is expressed in cardiomyocytes but its changes have not been detected in the plasma of patients with H hypertension; the miRNAs used in the study have a relevant role in different physiological processes of H hypertension as well as in pathogenic processes.

### 2.5. miRNA Analysis

Total RNA was extracted from serum using the Trizol method. Next, 20 *μ*l of cDNA was synthesized by reverse transcription using the Thermo-fermentedfermented reverse transcription polymerase reaction kit. 20 *μ*l of PCR reaction system was configured according to the instructions of QIAGEN's QuantiNova SYBR Green Dye Method PCR kit, followed by 2-3 wells of quantitative PCR reactions using a fluorescent quantitative U6 snRNA which was used as an internal reference. MiRNA-21 sequence was 5′-TCAGCCAACACCAGTCGATGGTGCAGGGTCCGAGGT-3′; the miR-29b sequence is 5′-ACAGCAATTAGCACCATTTGAATATGCTTCTTCTCGTCTCTGTGTC-3′; the miR-199a sequence is 5′- CCGAGACCCAGTGTTCAGACTACCAGTGCGTGTCGTGGAGT-3′; the U6 sequence is 5′-CAGCACATATACTAAAATTGGAACGACGAATTTGCGTGTCATCC-3′. The above miRNA and U6 sequences were designed and synthesized by Shanghai GenePharma Co., Ltd. The expression of the above miRNAs was determined under the following conditions: 95°C for 2 minutes, followed by 45 cycles of 95°C for 30 seconds and 60°C for 20 seconds. The data were analyzed using quantitative rotor gene software. The relative expression levels of miR-21, miR-29b, and miR-199a were calculated using the 2^−ΔΔCT^method.

### 2.6. Statistical Analysis

All data were tested for normality (Kolmogorov–Smirnov). Continuous variables were described by $\bar{X}$ ±SD. categorical variables were described by frequencies and percentages. For normally distributed data, one-way analysis of variance (ANOVA) was used for comparisons of multiple groups (≥3), and the LSD test was used for two-by-two comparisons. The Kruskal–Wallis test was used for comparison of non-normally distributed data. Statistical data were compared using *χ*^2^ test. Pearson correlation was used to explore the relationship between miRNA and hcy, systolic blood pressure, and diastolic blood pressure. Logistic regression was used to analyze the relationship between target miRNA and hcy. *P* < 0.05 was considered to be statistically significant. SPSS software version 25.0 was performed for the above statistical analysis.

## 3. Result

### 3.1. Baseline Characteristics of Patients with Type H Hypertension

The baseline characteristics of participants in four groups are shown in Table 1. There were differences in gender distribution, age, BMI, and smoking between the four groups (*P* < 0.05). And there were statistically significant differences in hcy, systolic blood pressure, and diastolic blood pressure between the groups. Among them, there were statistically significant differences between the H-type hypertension group and the control group in age, hcy level, mean systolic blood pressure, and mean diastolic blood pressure; between the simple hypertension group and the normal control group in age, BMI, mean systolic blood pressure, and mean diastolic blood pressure; and between the simple hyperhomocysteine group and the normal control group in hcy level and mean systolic blood pressure (*P* < 0.05).

### 3.2. Expression Levels of miR-21, miR-29, and miR-199 among the Four Groups

**Figure 1:**
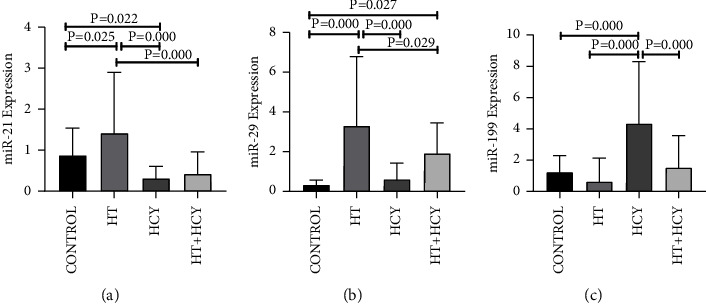
Plots of the expression levels of miR-21, miR-29, and miR-199 in the four groups. The results of multiple comparisons of (a) miR-21, (b) miR-29, and (c) miR-199 in subjects in CONTROL, HT, hcy, and HT + hcy groups. Data are shown as $\bar{X}$ ±SD. *Pvalues* were generated by one-way ANOVA followed by LSD post hoc multiple comparison test. *P* < 0.05 or *P* < 0.001 was considered significant.

As shown in Table 2, there were significant differences in the expression levels of miR-21, miR-29, and miR-199 among the four groups. As shown in Figure 1(a), the expression of miR-21 was significantly higher in the HT group than in the other three groups (*P* < 0.001). Furthermore, the difference was statistically significant in the HCY group compared with the CONTROL group (*P* < 0.001), and the expression of miR-21 was lower in the HCY group than in the CONTROL group (0.32 ± 0.29, 0.88 ± 0.66, *P* = 0.022). MiR-29 was expressed higher in the HT group than in the other three groups (*P* < 0.001), and the expression level of miR-29 was higher in the HT+HCY group compared with the CONTROL group (1.91 ± 1.54, 0.34 ± 0.24, *P* = 0.027) (Figure 1(b)). The expression of miR-199 was higher in the patients with pure hyperhomocysteinemia than in the other three groups, and there was a significant difference (*P* < 0.001) (Figure 1(c)).

### 3.3. Baseline Characteristics among MTHFRC677T Genotypes

As shown in Table 3, the differences in systolic and diastolic blood pressure between the genotypes of MTHFRC677T were statistically significant. There were no differences in sex, age, BMI, and hcy.

### 3.4. Association of MTHFRC677T Gene Polymorphism with Serum miR-199, miR-29, and miR-21 Expression

The HHcy patients with different genotypes of MTHFR (CC, CT, TT) were grouped according to each genotype, and the miRNA expression as well as the changes in blood pressure and hcy in each genotype was analyzed (Table 4). As shown in Table 2, in the CC genotype, there were statistically significant differences in the expression of hcy levels, miR-21, and miR-199 in the HHcy patients and the normal individuals. Moreover, the expression of miR-21 was lower in the HHcy patients than in the normal patients (0.29 ± 0.25 vs. 0.99 ± 1.23, *P* = 0.005), while the HHcy patients had higher miR-199 expression levels than the healthy individuals (3.35 ± 2.83 vs. 0.89 ± 1.61, *P* = 0.002). The expression of miR-21 was lower in the HHcy patients than in the normal individuals in the CT genotype (0.43 ± 0.53 vs. 1.44 ± 1.16, *P* = 0.001), and miR-199 expression was higher in the HHcy patients than in the normal individuals (1.84 ± 1.82 vs. 0.92 ± 1.00, *P* = 0.048) while no such differences were found in the TT genotype.

### 3.5. Association of Serum miR-199, miR-29, miR-21, and MTHFRC677T Gene Polymorphisms with H-type Hypertension

**Figure 2:**
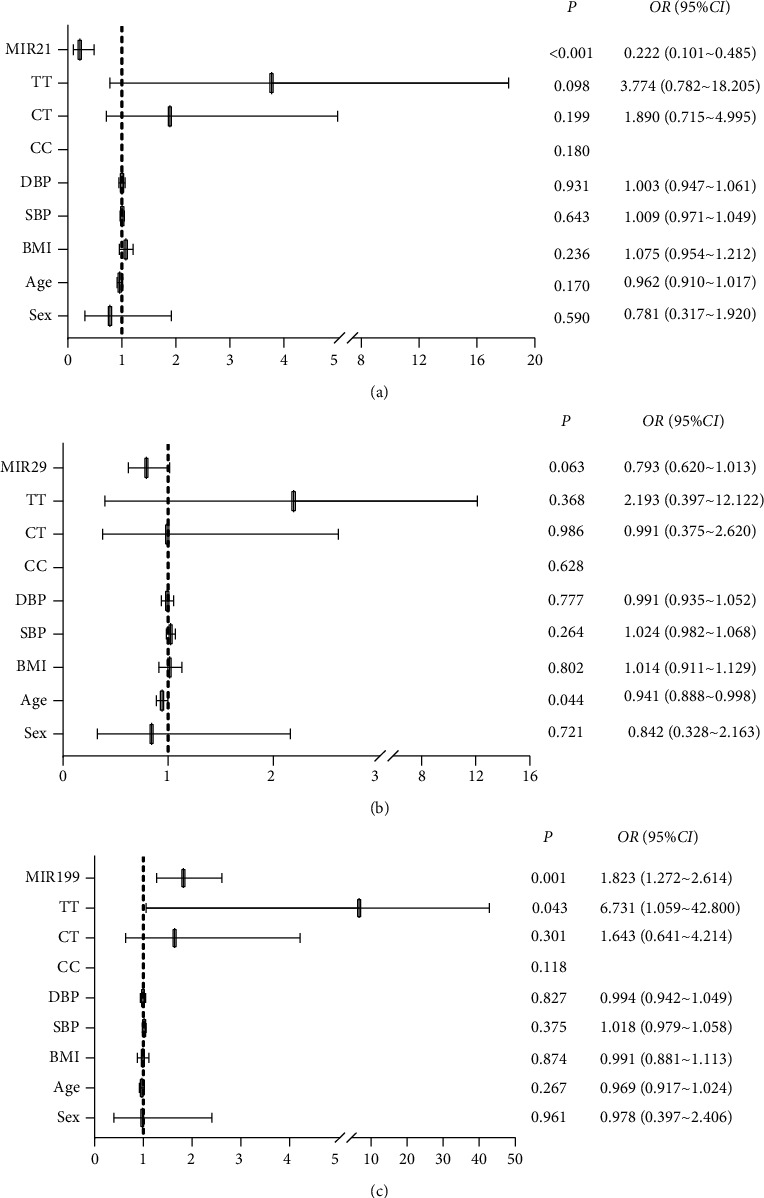
Forest plots of multivariate logistic regression analysis of the association between HCY patients and miR-21, miR-29, and miR-199 expression levels. The logistic regression analysis respectively adjusted sex, age, BMI, systolic blood pressure, diastolic blood pressure, and MTHFR C677T gene polymorphism. *P* < 0.05 or *P* < 0.001 was considered as significant.

Correlation analysis showed that miR-21 was negatively correlated with hcy. miR-29 was positively correlated with systolic and diastolic blood pressure. miR-199 was positively correlated with hcy and negatively correlated with systolic and diastolic blood pressure (Table 5). Logistic regression analysis (Figure 2) showed that after adjusting for gender, age, BMI, systolic blood pressure, diastolic blood pressure and MTHFRC677T gene polymorphism, miR-21 expression level was significantly and negatively correlated with hyperhomocysteinemia prevalence (OR = 0.222, 95% CI: (0.101–0.485), *P* < 0.001), and each unit increase in miR-199 expression was associated with an increased risk of hyperhomocysteinemia (OR = 1.823, 95%CI: (1.272–2.614), *P* = 0.001). And the miR-29 did not correlate with HHcy (*P* = 0.063) shown in Figure 2(b).

## 4. Discussion

The present study is the only one to demonstrate an association between miR-21, miR-29, and miR-199 and H-type hypertension, especially hyperhomocysteinemia in the Kazakhs of Xinjiang, China. In this study, all subjects were come from the Kazakh cohort in Xinjiang and did not receive any intervention, and we had complete archived serum samples. We demonstrated the association of miR-21, miR-29, and miR-199 with H-type hypertension, considering whether the MTHFRC677T gene polymorphism affects miRNA expression and thus its association with H-type hypertension.

In the present study, MiR-21 was negatively correlated with serum hcy. However, few studies reported the association between MiR-21 and hcy. miR-21 antagonists upregulated cystathione-*β*-synthase (CBS) and cystathione-*γ*-cleavage enzyme (CSE) expression in an vitro assay [15]. CBS and CSE catalyze the removal of hcy through its conversion to cysteine. It has been suggested that CBS activity may be positively correlated with hcy levels. Therefore, our findings remain consistent with the above current theory [16, 17].

Correlation analysis showed that miR-29 was positively correlated with both systolic blood pressure and diastolic blood pressure. In multiple studies [9, 18], miR-29b has been shown to act as a biomarker of hypertension, but the relationship with hcy is unclear. HHcy has been shown to affect blood-brain barrier (BBB) integrity through upregulation of matrix metalloproteinases (MMPs plays a key role in tumor invasion and metastasis by degrading various protein components in the extracellular matrix (ECM) and destroying the histological barrier to tumor cell invasion), where BBB integrity is mediated by miR-29b through regulation of DNMT3B (DNA methyltransferases 3B) which regulates the levels of MMP9 (matrix metalloproteinases 9, which is the largest molecular weight enzyme in the matrix metalloproteinase family) [19]. The author also found that treatment with homocysteine (50–200 *μ*M) increased miR-29b levels as well as MMP9 expression and activity in mouse brain endothelial cells. However, miR-29b was not shown to be correlated with hcy levels in this study, so the results may still be controversial. This may be due to different disease models.

We demonstrated that miR-199 expression levels were positively correlated with hcy and negatively correlated with hypertension. Previous studies reported miR-199a-5p as a circulating serum marker of hypertension [20, 21]. Upregulation of miR-199a-5p has been reported in the lung tissue from mouse and rat models of pulmonary artery hypertension (PAH), which also identified SMAD3 (which plays a key role in transmitting TGF-*β* signals from cell surface receptors to the nucleus) as a target gene of miR-199a-5p [10]. This link is important because defects in SMAD-dependent signaling have been linked to hypertension and cardiac pathologies, primarily through their association with TGF-*β* pathways [22]. This is not in line with the negative association of miR-199 with blood pressure in the present study. We speculate that the inconsistent results may be due to different disease types, different experimental models, different patient ethnicities, and different disease stages.

However, a retrospective human study found that the downregulation of miR-199a-5p in maternal peripheral blood was associated with gestational hypertension, preeclampsia, and intrauterine growth restriction. The authors also concluded that dysregulation of miR-199a-5p may be associated with epigenetic alterations due to pregnancy-related complications [23]. This finding is relevant to our current study because MTHFR is a key enzyme in the Hcy metabolic pathway. Meanwhile, it may affect DNA methylation [20]. It has been shown that the methylated levels of NOS3 (nitric oxide synthase 3, which is mainly distributed in the endothelium of coronary vessels and cardiac luminal surfaces to catalyze the production of nitric oxide (NO)) and GNA12 (G Protein Subunit Alpha 12, which is an important component in the cell signaling pathway) loci differ significantly between individuals with CC and TT genotypes, and this study also provides some preliminary data on the mechanism of association between MTHFR and blood pressure [24]. In addition, the expression of miRNAs is also regulated by epigenetic mechanisms, including DNA methylation, RNA modifications, and histone modifications [25]. SAM (S-adenosylmethionine) is a universal methyl donor for histone and DNA methylation, and its specificity is involved in the epigenetic maintenance of cancer cells. The folate cycle promotes SAM production. MTHFR is a key enzymes for folate metabolism and methyl donor SAM production [26], and knockdown of MTHFR significantly inhibits GC cell proliferation [27]. miR-22 overexpression reduces endogenous SAM levels by inhibiting MTHFR and inducing P16, PTEN, and RASSF1A (they are anticancer genes) hypomethylation [28]. Therefore, we consider that the polymorphism of MTHFRC677T can affect the expression of our target miRNAs, and even the two may interact with each other to form a feedback network. In a mouse model of ovalbumin-induced allergic rhinitis [29], miR-199 is significantly elevated and is involved in the pathogenesis of autosomal recessive disorder disease through the miR-199-3p-Dnmt3a-STAT3 signaling pathway. Dnmt3a methylates and regulates the expression of a large number of genes [30]. The Auto-CM analysis also linked low hcy levels to high promoter methylation of genes required for DNA methylation reactions (DNMT1, DNMT3A, DNMT3B, and MTHFR). Auto-CM analysis linked low hcy levels to high promoter methylation of genes required for the DNA methylation response (DNMT1, DNMT3A, DNMT3B, and MTHFR). Folic acid can affect DNA methylation levels by regulating proteins required for the DNA methylation response, and MTHFR is a key protein for the interconversion of folate derivatives required for the DNA methylation response [31]. Previous studies in humans showed that the MTHFR promoter shows interindividual variability in DNA methylation patterns that correlate with circulating folate, vitamin B12, and/or hcy levels [32]. It has been shown that the association of miR-199a-5p with blood pressure was only evident in the TT group of patients [11]. Indeed, aberrant hypermethylation and expression of DNA methylation-related genes have been reported in patients with the variant MTHFR 677TT genotype [20, 33], and epigenetic mechanisms are known to be involved in hypertension [34, 35]. In addition, it is noteworthy that there is evidence from COPD studies that miR-199a-5p is indeed regulated by its promoter methylation status [36]. The authors also found that the expression of miR-199a-5p in patients with MTHFR 677TT genotype may be influenced by aberrant DNA methylation [11].

The presence of the T allele of the MTHFRC677T polymorphism corresponds to higher serum miR-21 expression in a patient model of colon cancer. This author also proposed that serum miR-21 expression correlates with folate status and related genetic status [37], and that folate deficiency is known to be an important causative agent of HHcy [38]. miR-29b belongs to the miR-29 family and targets DNMTs and TETs (ten-eleven translocations, which is an important enzyme in the process of DNA demethylation), thereby affecting DNA methylation. During early porcine embryonic development, miR-29b inhibitor increased overall genomic DNA methylation levels by upregulating DNMT3A/B and TET1 and downregulating TET2/3 [39]. Similarly, miR-29 inhibition resulted in DNMT3A upregulation and genomic methylation with silencing of the tumor suppressor PTEN [40]. Although there was no statistical difference in the expression of miR-21, miR-29, and miR-199 among patients with CC, CT, and TT genotypes in this study, miR-199a expression in patients with 677TT genotype may also be affected by aberrant DNA methylation in our sample due to the small sample size in this study. Therefore, different genotypes may affect the epigenetic mechanisms of miRNAs, especially DNA methylation, and thus their expression. If the epigenetic regulation of miR-21, miR-29, and miR-199 is similarly affected, then we deduced that different genotypes of MTHFRC677T may affect the miR-21, miR-29, and miR-199 association with hcy, but the exact mechanism is unknown to us.

Kazakhs have distinctive ethnic aggregation characteristics, living environment, and genetic background so they constitute extremely valuable genetic information. The prevalence of hypertension among Kazakhs was 36.5% in our group's previous survey, which was significantly higher than that of local Han Chinese (28.9%) and Uyghurs (25.7%) [41]. Therefore, it is important to clarify the possible pathogenesis of H-type hypertension to prevent and treat hypertension in this ethnic group. In addition, it has been shown that ethnic differences may be a factor affecting miRNA expression. In the present study, miR-21 was significantly expressed in Kazakh hypertensive patients. In agreement with our results, Hu et al. showed that Han Chinese patients with pulmonary hypertension had higher circulating miR-21 levels than healthy controls. On the other hand, HHD patients had significantly lower miR-29 levels. However, miR-29 was positively correlated with SBP and DBP in our study, which is consistent with the findings of Luo et al. [9]. In addition, there are fewer studies on miR-199 expression in patients with H-type hypertension. It is noteworthy that the negative association of miR-199a-5p with blood pressure in patients with the MTHFR 677TT genotype found in the study of Lynch et al. is consistent with the results of our current study [11]. Whether the inconsistency of the above results is due to ethnic differences needs further validation.

### 4.1. Limitations

The conducted study incorporates several limitations. First, only a small sample was investigated in this study, so further confirmation in a larger prospective study at all stages of the disease is needed to clearly show that miR-21, miR-29, and miR-199 are true markers in patients with H-type hypertension. Second, there were fewer patients with MTHFR 677TT genotype in this study and more subjects should be recruited and similarly evaluated to further understand the relationship between the above-mentioned 3 miRNAs and MTHFR gene polymorphisms. Third, considering that there may be other microRNAs involved in H-type hypertension, a more comprehensive approach, such as microRNA arrays, may be needed to identify new targets other than miR-21/-29b/-199a. Last, the mechanism of miRNA action was not explored in this study, and the hypothesized potential mechanism is based on previous studies, and further experimental studies or raw letter analysis are needed to explore the unknown pathways of miR-21, miR-29, and miR-199 in H-type hypertension.

## 5. Conclusion

In summary, this study demonstrated that miR-21 was significantly negatively correlated with serum hcy levels, miR-199 expression levels were significantly positively correlated with serum hcy and negatively correlated with blood pressure, and correlation analysis showed that miR-29 was negatively correlated with both systolic and diastolic blood pressure, which has great clinical significance because local targeting of these miRNAs in patients with H-type hypertension may be a beneficial therapeutic strategy in the future. This study linked MTHFRC677T gene polymorphism with miRNA expression. Although miRNA expression did not differ between genotypes, gene polymorphisms are likely to disrupt the expression of transcription factors critical to miRNA transcription, indirectly affecting miRNA expression. MiR-21, miR-29b, and miR-199a could be used as biomarkers to provide clues to the underlying pathogenesis of H-type hypertension.

## Figures and Tables

**Table 1:** Baseline characteristics of the four groups.

	HT + hcy (*n* = 100)	HT (*n* = 100)	hcy (*n* = 100)	NC (*n* = 100)	*F*/*χ*^2^	*P*
Gender						
Male	61	39	59	52	11.906	0.008^*∗*^
Female	39	61	41	48		
Age (years)	53.54 ± 13.52^a^	49.40 ± 10.27^a^	42.09 ± 12.31	41.86 ± 10.10	24.308	<0.001^*∗*^
BMI (Kg/m^2^)	25.76 ± 5.03	26.56 ± 4.25^a^	24.55 ± 3.35	24.91 ± 4.27	4.543	0.004^*∗*^

Smoking						
No	60	83	63	73	15.486	0.001^*∗*^
Yes	40	17	37	27		

Drinking						
No	86	91	88	91	1.839	0.607
Yes	14	9	12	9		

Family history of hypertension						
No	58	47	57	54	2.979	0.395
Yes	42	53	43	46		
HCY (umol/l)	22.69 ± 12.94^a^	12.18 ± 1.73	19.38 ± 6.46^a^	11.01 ± 2.29	58.404	<0.001^*∗*^
SBP (mm·Hg)	160.64 ± 21.28^a^	158.36 ± 18.91^a^	121.58 ± 9.74^a^	126.91 ± 8.93	170.514	<0.001^*∗*^
DBP (mm·Hg)	94.50 ± 12.95^a^	92.59 ± 13.15^a^	77.20 ± 6.89	76.37 ± 8.08	83.229	<0.001^*∗*^

^*∗*^ *P* < 0.05 and ^*∗∗*^*P* < 0.001 were considered significant; compared with normal control group. ^a^*P* < 0.05.

**Table 2:** Comparison of the expression levels of miR-21, miR-29, and miR-199 in the four groups.

	HT + hcy group (*n* = 30)	HT group (*n* = 30)	hcy group (*n* = 30)	NC group (*n* = 30)	*P*
MiR-21	0.42 ± 0.54	1.42 ± 1.48	0.32 ± 0.29	0.88 ± 0.66	<0.001^*∗∗*^
MiR-29	1.91 ± 1.54	3.30 ± 3.48	0.60 ± 0.82	0.34 ± 0.24	<0.001^*∗∗*^
MiR-199	1.52 ± 2.05	0.65 ± 1.50	3.51 ± 2.69	1.24 ± 1.06	<0.001^*∗∗*^

^*∗*^ *P* < 0.05 and ^*∗∗*^*P* < 0.001 were considered significant.

**Table 3:** Comparison of baseline characteristics among the four groups of MTHFRC677T genotypes.

	CC	CT	TT	*P*
Sex				
Female	24	25	10	0.839
Male	36	23	2	
Age (years)	45.88 ± 12.81	47.82 ± 12.08	46.95 ± 13.51	0.357
BMI (Kg/m^2^)	25.29 ± 4.22	25.90 ± 4.45	24.66 ± 4.15	0.192
SBP (mmHg)	138.78 ± 21.80	145.72 ± 25.43	143.43 ± 24.42	0.021^*∗*^
DBP (mmHg)	83.47 ± 11.87	87.60 ± 15.28	84.85 ± 13.70	0.017^*∗*^
HCY (*μ*mol/l)	15.63 ± 8.98	16.62 ± 9.05	18.62 ± 6.65	0.116

^*∗*^ *P* < 0.05 and ^*∗∗*^*P* < 0.001 were considered significant.

**Table 4:** Comparison of miRNA, blood pressure, and hcy levels of different genotypes of MTHFRC677T.

	CC		*P*	CT		*P*	TT		*P*
	HHcy (*n* = 28)	Control (*n* = 32)		HHcy (*n* = 23)	Control (*n* = 25)		HHcy (*n* = 9)	Control (*n* = 3)	
MiR-21	0.29 ± 0.25	0.99 ± 1.23	0.005^*∗*^	0.43 ± 0.53	1.44 ± 1.16	0.001^*∗∗*^	0.38 ± 0.50	0.62 ± 0.48	0.497
MIR-29	1.03 ± 1.66	2.60 ± 3.40	0.081	1.50 ± 0.95	2.00 ± 2.95	0.500	1.59 ± 1.96	0.33 ± 0.12	0.176
MIR-199	3.35 ± 2.83	0.89 ± 1.61	0.002^*∗*^	1.84 ± 1.82	0.92 ± 1.00	0.048^*∗*^	2.31 ± 3.30	1.30 ± 0.43	0.691
SBP (mmHg)	137.04 ± 20.79	143.08 ± 16.71	0.217	148.63 ± 26.11	141.80 ± 20.80	0.319	151.33 ± 33.93	132.67 ± 4.16	0.379
DBP (mmHg)	83.77 ± 12.68	86.31 ± 10.68	0.402	87.09 ± 18.30	84.46 ± 15.14	0.589	84.00 ± 18.91	76.17 ± 8.61	0.513
Hcy(*μ*mol/l)	23.71 ± 16.77	11.53 ± 1.98	0.001^*∗∗*^	19.84 ± 7.17	11.10 ± 2.66	<0.001^*∗*^	17.80 ± 3.34	10.80 ± 1.35	0.006^*∗*^

^*∗*^ *P* < 0.05 and ^*∗∗*^*P* < 0.001 were considered significant.

**Table 5:** Correlation of miR-21, miR-29, and miR-199 with hcy and blood pressure.

	MiR-21	MIR-29	MIR-199	SBP	DBP
HCY	−0.354^*∗∗*^	0.102	0.322^*∗∗*^	0.033	0.020
MIR-21		−0.003	0.186	-0.005	0.064
MIR-29			−0.372^*∗∗*^	0.599^*∗∗*^	0.448^*∗∗*^
MIR-199				−0.390^*∗∗*^	−0.318^*∗∗*^
SBP					0.721^*∗∗*^

^*∗*^ *P* < 0.05 and ^*∗∗*^*P* < 0.001 were considered significant.

## Data Availability

Data cannot be shared publicly. Datasets are available from the corresponding author by request.

## Abbreviations

HT: Hypertension
HHcy: Hyperhomocysteinemia
hcy: Homocysteine
MTHFR: Methylenetetrahydrofolate reductase
miRNAs: MicroRNAs
LVMI: Left ventricular mass index
SMAD 4/3: Recombinant mothers against decapentaplegic homolog 4/3
CBS: Cystathione-*β*-synthase
CSE: Cystathione-*γ*-cleavage enzyme
BBB: Blood-brain barrier
MMP: Matrix metalloproteinase
DNMT 3B/3A/1: DNA methyltransferases 3B/3A/1
NOS3: Nitric oxide synthase 3
GNA12G: Protein Subunit Alpha 12
SAM: S-Adenosylmethionine
P16: Multiple tumor suppressor 1/MTS
PTEN: Phosphate and tension homology deleted on chromosome ten
RASSF1A: Ras association domain family 1A
TET 1/2: Ten-eleven translocation 1/2.
